# Mucormycosis-induced upper gastrointestinal ulcer perforation in immunocompetent patients: a report of two cases

**DOI:** 10.1186/s12876-021-01881-8

**Published:** 2021-08-03

**Authors:** Hongyun Huang, Lang Xie, Zheng Zheng, Hanhui Yu, Lingjing Tu, Chunhui Cui, Jinlong Yu

**Affiliations:** 1 The Second School of Clinical Medicine, Southern Medical University, Guangzhou, 510515 Guangdong China; 2 Department of General Surgery of Zhujiang Hospital, Southern Medical University, Guangzhou, 510280 China

**Keywords:** Gastrointestinal bleeding, Gastrointestinal perforation, Gastrointestinal mucormycosis, Mucor, Mucormycosis

## Abstract

**Background:**

Gastrointestinal mucormycosis (GIM) is a rare, opportunistic fungal infection with poor prognosis. Clinically, it is difficult to diagnose GIM owing to its nonspecific clinical symptoms and poor suspicion. The estimated incidence of GIM is inaccurate, and most cases are diagnosed accidentally during surgery or upon postmortem examination. GIM usually occurs in patients with immune deficiencies or diabetes. Here, we report two cases of immunocompetent young patients with GIM who had good prognosis after treatment. Compared to other case reports on GIM, our cases had unusual infection sites and no obvious predisposing factors, which make it important to highlight these cases.

**Case presentation:**

The first case was that of a 16-year-old immunocompetent boy who was admitted with gastrointestinal bleeding and perforation due to a gastric ulcer. Strategies used to arrest bleeding during emergency gastroscopy were unsuccessful. An adhesive mass was then discovered through laparoscopy. The patient underwent type II gastric resection. Pathological examination of the mass revealed bacterial infection and GIM. The second case was of a 33-year-old immunocompetent woman with a recent history of a lower leg sprain. The patient subsequently became critically ill and required ventilatory support. After hemodynamic stabilization and extubation, she presented with hematemesis due to exfoliation and necrosis of the stomach wall. The patient underwent total gastrectomy plus jejunostomy. The pathology results revealed severe bacterial infection and fungal infection that was confirmed as GIM. The patient fully recovered after receiving anti-infective and antifungal treatments.

**Conclusions:**

Neither patient was immunosuppressed, and both patients presented with gastrointestinal bleeding. GIM was confirmed via pathological examination. GIM is not limited to immunocompromised patients, and its diagnosis mainly relies on pathological examination. Early diagnosis, timely surgical treatment, and early administration of systemic drug treatment are fundamental to improving its prognosis.

**Supplementary Information:**

The online version contains supplementary material available at 10.1186/s12876-021-01881-8.

## Background

Mucormycosis is a rare, life-threatening, angioinvasive fungal infection that accounts for 10% of all mycotic infections [1]. It is caused by the fungi in the genus *Rhizopus* of the family *Mucoraceae*. Based on clinical presentation and anatomical site of infection, mucormycosis is categorized into six clinical types as follows: (1) rhino cerebral, (2) pulmonary, (3) cutaneous, (4) gastrointestinal, (5) disseminated, and (6) miscellaneous (brain, bones, mediastinum, trachea, kidneys, etc.) [2]. The fungi can invade blood vessels and bowel walls, resulting in sepsis, peritonitis, massive gastrointestinal hemorrhage, and bowel perforation, which is the most common cause of death. Despite the intensive use of antifungal therapies and surgical debridement, the mortality rate of adult patients ranges from 20 to 100% [3]. Overall, the intestines (71.6%) (large intestine, 43.2%; small intestine, 28.4%) are the most affected organs, followed by the stomach (33.0%) [4], accounting for 4%–7% of all documented cases with a mortality rate of 85% due to bowel perforation and upper gastrointestinal hemorrhage [5]. However, only 25% of the reported cases are diagnosed antemortem [5].

Herein, we report a case of gastrointestinal mucormycosis (GIM) in a 16-year-old boy with gastrointestinal bleeding and perforation due to a large gastric ulcer and a case of a 33-year-old woman suffering from posterior gastric wall necrosis due to gastric infection with mucor after long-term treatment of limb trauma. Neither of these patients had any identified risk factors for mucormycosis, making these cases rare and worthy of reporting.

## Case presentation

### Case 1

**Fig. 1:**
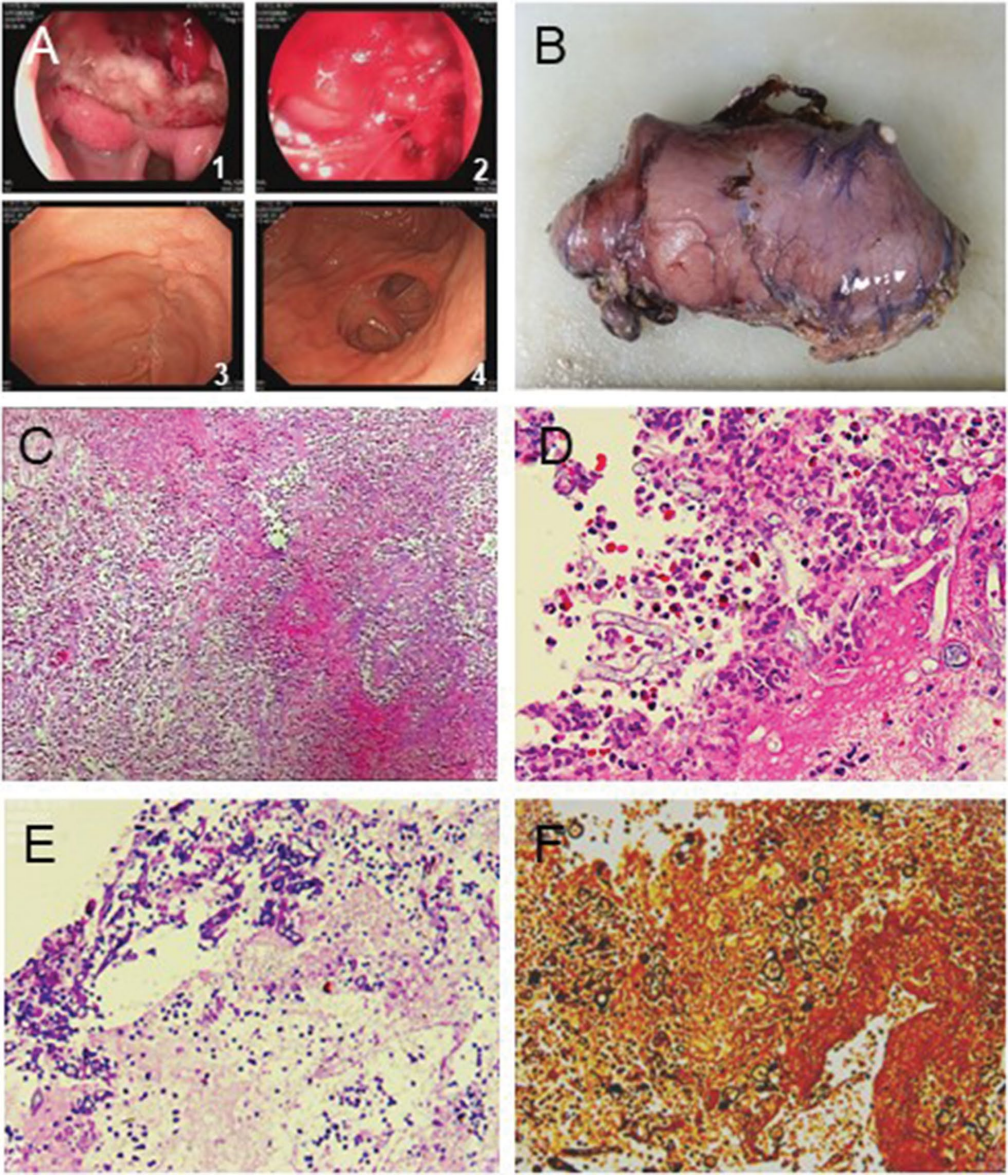
Microscopic findings of resected stomach of case 1. **a1**, **a2** The first-time gastroscopy; **a3–a4** Gastroscopy after the surgical treatment; **b** Surgically removed tissue; **c** Inflammatory infiltration (Hematoxylin & Eosin stain, × 100); **d**–**f** Mucormycosis (**d** Hematoxylin & Eosin stain; **e** Periodic Acid Schiff stain; **f** Gomori methenamine-silver stain. × 400)

A 16-year-old boy was admitted to the hospital with symptoms of epigastric discomfort, sudden onset of hematemesis, and hemodynamic instability. The patient was normally healthy with no similar diseases, other infectious diseases, or history of drinking. His systolic blood pressure was 81 mmHg, and heart rate (HR) was 132 bpm. The results of laboratory tests were as follows: hemoglobin (HGB), 58 g/L; white blood cells (WBC), 10.93 × 10^9^/L; and neutrophils (NEUT), 62.4%. Liver function, renal function, and electrolyte levels were normal. The patient received anti-shock therapy and was infused with two units of concentrated red blood cells. Emergency gastroscopy revealed that heavy bleeding was existed in patient’s upper gastrointestinal (Fig. 1, a1, a2). A titanium clamp was placed under the endoscopy, and norepinephrine was sprayed over the ulcer to control bleeding. Due to the ineffectiveness of these hemostatic methods, the patient was quickly transferred to the operating room. Laparotomy revealed a 3 cm × 5 cm mass at the gastroduodenal junction. The mass had dense inflammatory adhesions with the surrounding area and the pancreas (Fig. 1b, Additional file 1: Figure S1A). Due to local scar tissue, edema of the duodenal wall, and high risk of poor healing of the duodenal stump after subtotal gastrectomy, a duodenal fistula was placed on the stump of the duodenum with a complete type II gastric resection, and a drainage tube was placed nearby. After the operation, the patient’s HGB level was 57 g/L, and there was no further bleeding. Postoperative pathological examination revealed a chronic gastroduodenal ulcer with necrosis, perforation, and mycosis (inclined to mucor), complicated with bacterial infection (Fig. 1, c–f). On the third day postoperation, the patient developed fever, and 50 mL of dark green fluid was drained from the abdominal drainage tube. The patient improved after receiving imipenem/cilastatin sodium. Postoperative blood fungal tests were negative. Therefore, antifungal treatment was not required. Two weeks after discharge, gastrointestinal angiography showed the formation of a partially stable sinus; the duodenostomy and abdominal drainage tubes were removed, and the patient recovered well (Fig. 1, a3, a4). After 5 months, further fungal tests were negative, and the prognosis was good.

### Case 2

**Fig. 2:**
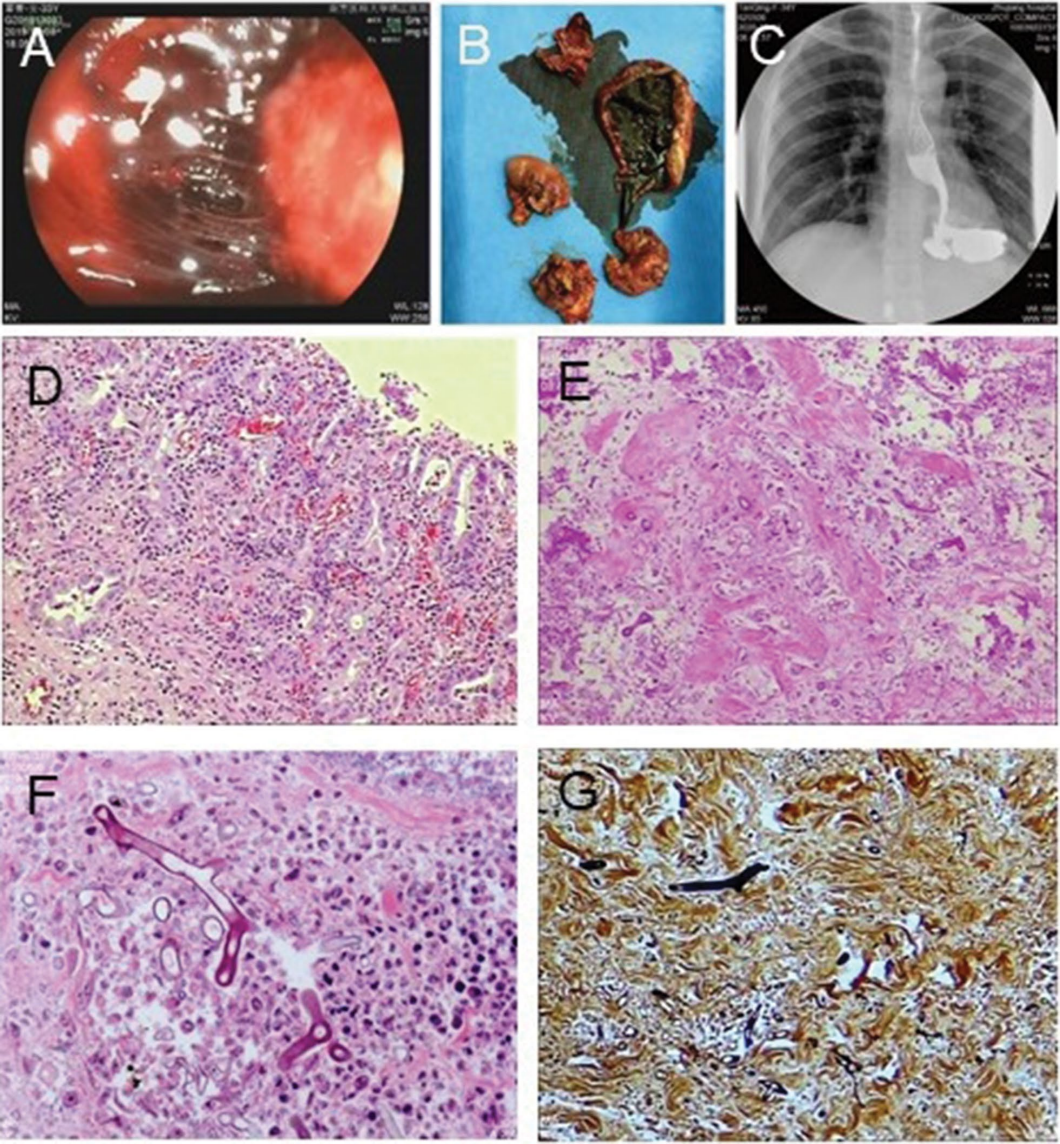
Microscopic findings of resected stomach of case 2. **a** Perforation was notice by the endoscopes; **b** the specimen of total gastric necrosis tissue during the first stage operation; **c** the digestive tract radiography after esophagojejunostomy; **d** Gastric mucosa inflammation (Hematoxylin & Eosin stain, × 100); **e**–**g** Mucormycosis (**e** Hematoxylin & Eosin stain, × 100; **f** Periodic Acid Schiff stain, × 400; **g** Gomori methenamine-silver stain, × 400)

A 33-year-old woman suffered a sprained left leg in a cycling accident. Before admitted to hospital with gastric necrosis caused by traumatic, patient has good nutritional status and there are no tobacco, alcohol and any bad diet habits. Her medical history consisted oral medications, topical Chinese herbal drugs, and cupping at a local clinic. However, her left leg gradually became edematous, and there was no remission after symptomatic treatment of the affected limb with cold packs. Around the same time the edema was significantly worse, the patient also developed an itchy abdominal rash that gradually spread to her entire body. She then visited a local hospital for further treatment. The results of routine laboratory tests were as follows: WBC, 12.06 × 10^9^/L; NEUT, 91.7%; platelets (PLT), 150 μg/mL, arterial blood gas analysis: pH, 7.217; base excess (BE), − 19.1 mmol/L; and bicarbonate levels, 6.7 mmol/L. Anti-allergic and anti-inflammatory treatment were administered based on the diagnosis. Due to the presence of severe infection with metabolic acidosis, the patient developed dizziness and shortness of breath. Her blood pressure was 45/25 mmHg, and HR was 140 bpm. The patient was transferred to the intensive care unit (ICU) and was administered emergency treatment to stabilize her vitals. After 7 days, the patient was transferred to our hospital for further treatment. Her vital signs were as follows: temperature, 38.2 °C; HR, 134 bpm; and blood pressure, 88/59 mmHg (maintained using norepinephrine 1 μg/kg/min). The patient was sedated and given ventilatory support. She was diagnosed with septic shock, multiple organ dysfunction syndrome, and ulceration of the left calf. On admission, her laboratory tests were as follows: WBC, 28.8 × 10^9^/L; HGB, 68 g/L; PLT, 29 g/L; urea, 18.27 mmol/L; creatinine, 149.57 μmol/L; albumin, 17.4 g/L; D-dimer > 20.0 mg/L; and troponin T, 1.130 μg/L. Cardiac color Doppler ultrasound revealed that cardiac contractions during systole were weak, with an ejection fraction of approximately 35%. After continuous treatment with diuretics, antibiotics, ventilator therapy, blood transfusion, and nutritional support, her vital signs stabilized. The patient underwent escharectomy of the left lower limb twice. Due to long-term antibiotics and other symptomatic treatment, the patient was removed from the ventilator 16 days after the injury. After extubation, her vital signs were stable. Nine days after extubation, hematemesis occurred, accompanied by chills and fever. Emergency gastroscopy revealed a large blood clot in the gastric cavity, leading to the diagnosis of upper gastrointestinal bleeding accompanied with upper gastrointestinal perforation (Fig. 2a). The patient underwent immediate laparotomy. During the operation, numerous blood clots were found in the stomach cavity (Fig. 2b). The entire posterior wall of the mucosa and muscle layers of the stomach were exfoliated and necrotic, with local abscess formation and inflammatory edema of the local tissue. Considering the hemorrhagic shock, severe infection, and high risk of one-stage anastomoses, total gastrectomy plus jejunostomy was performed (Additional file 1: Figure S1B). and the patient was transferred to the ICU after surgery. After esophagojejunostomy, the digestive tract radiography shown that there were no contrast agent extravasation and stenosis in upper digestive tract (Fig. 2c). Blood investigations indicated severe infection, fungal D-polysaccharides, and negative fungal antigens. Considering the severe infection, the patient was started on imipenem/cilastatin sodium plus teicoplanin. Postoperative pathology showed there were acute suppurative inflammation with ulcer formation and mycosis in the stomach (Fig. 2, d–g). Antifungal treatment with amphotericin B was initiated, and the patient continually improved. After 1 month of treatment, the patient was discharged from the hospital without physical discomfort. After 8 months, the patient showed good prognosis without recurrence or other complications.

## Discussion and conclusions

Mucormycosis is a rare and well-recognized fungal infection that can occur in any part of the alimentary system and has a high mortality rate in immunocompromised patients. Over the past two decades, GIM has been increasingly reported in individuals without typical risk factors of immunosuppressive conditions or uncontrolled diabetes mellitus [1, 6]. These so-called health-associated GIM occur in immunized patients in ICUs after long-term hospitalization and major surgery. A previous study has reviewed cases of GIM in immunocompetent individuals with lower survival rates [7]. Patients were reportedly hospitalized for different underlying causes, such as trauma, septic shock, or respiratory symptoms, and the patients then developed upper or lower gastrointestinal bleeding. This is consistent with the symptoms reported in our case. Case 1 was of a 16-year-old adolescent boy who had chronic gastroduodenal ulcer with necrosis, perforation, and mycosis (inclined to mucor), complicated with bacterial infection. Case 2 was of a 33-year-old woman diagnosed with upper gastrointestinal bleeding complicated with upper gastrointestinal perforation. Therefore, we suggest that gastrointestinal bleeding should be considered as an early marker for the differential diagnosis of GIM. With perforation and massive bleeding in the digestive tract, patients’ condition often deteriorate rapidly due to septic shock and multiple organ failure. The clinical features of mucormycosis are extensive angioinvasion with vessel thrombosis and tissue necrosis. During fungal infection, normal endothelial cells may not be able to maintain their normal vascular tone and permeability. Simultaneously, reduced oxidative metabolites and cationic peptides of endothelial cells leads to phagocytosis of the dead fungus and further tissue damage [2]. Antifungal treatment alone is usually insufficient in controlling invasive mucormycosis due to angioinvasion and thrombosis of the vessels, which causes less optimal penetration of medications to the site of interest. The mortality rate of patients with GIM is quite high [7–9]. Hence, surgery to debulk the fungal infection with resection of all the infected necrotic tissue is often required, along with systemic antifungal treatment. Additionally, surgery should be performed before the patient is immunologically active [8, 9]. In our case, both patients underwent surgical excision of the lesion in time, thereby interrupting extensive blood invasion, and the patients recovered after active treatment and care. Therefore, timely surgical debridement and antifungal drugs are essential for the treatment of GIM. Currently, liposomal amphotericin B is the preferred agent with a favorable nephrotoxicity profile, superior central nervous system penetration, and greater efficacy [10]. In our case, imipenem/cilastatin sodium and amphotericin B were effective.

Accurately diagnosing GIM is challenging and is often delayed. The initial presentation of gastrointestinal lesions consists of abdominal pain, distention, fever, and diarrhea. If the infection extends into the lumen of the gut, it can lead to obstruction, ulceration, bleeding, or perforation. Radiologically, nodules and pleural effusion are reportedly common in mucormycosis, but the imaging findings are nonspecific [11]. However, a fungal culture is a gold standard for diagnosis. It can be used to confirm the diagnosis and allows the identification of precise genus and species [12–14]. In histopathological examination, the fungi show septate, broad, and non-septate hyphae (6–25 μm) that branch at right or obtuse angles [15]. In our study, GIM was diagnosed using postoperative pathological examination. Molecular testing with tissue samples can also confirm the histological diagnosis, such as in situ hybridization targeting 5S and 18S ribosomal RNA sequences [16, 17]. In addition, serum galactomannan (a fungal antigen found in the sera of infected patients) level test can be used for the early diagnosis of mucormycosis. Despite the available diagnostic methods, it is still imperative to establish early diagnosis and to commence systemic antifungal therapy and surgical intervention early, considering the high mortality rate associated with mucormycosis.

In the two successfully cured cases, although their infection route has not been confirmed, both patients benefited from early detection, timely surgery, and antimicrobial therapy. In case 1, the patient was infected via an unknown route, although it may be related to a long-term irregular diet. After the removal of most of the stomach, the patient’s condition improved and was discharged with no fungal recurrence. In case 2, the patient was infected due to a secondary infection with an immunocompromised condition. After surgical debridement and symptomatic treatment, the patient recovered. Based on our two cases, we suggest that endoscopy should be performed as early as possible when patients present with gastrointestinal symptoms. Due to acute onset, the two cases did not improve with treatment based on the preoperative examination. Fortunately, both immunocompetent patients underwent surgery to remove the lesions, received prompt systemic treatment, and had good outcomes.

Similar to the findings of a case report published by Abreu et al*.* [18], our positive patient outcomes suggest that increased reporting and awareness of fungal infections in immunocompetent adults may lead to early administration of antifungal drugs, early surgical treatment, and better outcomes. If fungal infection is suspected according to clinical diagnosis, surgical treatment could be considered to eradicate the primary lesion, which may increase the patient's chance of cure.

## Supplementary Information

**Additional file 1:Figure S1.** Intraoperative images. (A) Tissue adhesion and local hemostasis in case 1. (B) The second-stage esophageal jejunostomy of case 2.

## Data Availability

The datasets used and/or analyzed during the current study are available from the corresponding author upon reasonable request.

## Abbreviations

HR: Heart rate
GIB: Gastrointestinal bleeding
GIM: Gastrointestinal mucormycosis
HGB: Hemoglobin
WBC: White blood cells
NEUT: Neutrophils
ICU: Intensive care unit
PLT: Total platelet
