# Supplementary figures and images for: Mucormycosis-induced upper gastrointestinal ulcer perforation in immunocompetent patients: a report of two cases
Source: BMC Gastroenterol. 2021 Aug 3;21:311. doi: 10.1186/s12876-021-01881-8 (PMC8370051; doi:10.1186/s12876-021-01881-8)

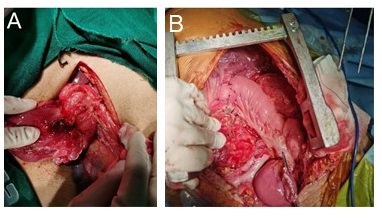

Supplement: Supplementary file 1 — Additional file 1:Figure S1. Intraoperative images. (A) Tissue adhesion and local hemostasis in case 1. (B) The second-stage esophageal jejunostomy of case 2. [file 12876_2021_1881_MOESM1_ESM.jpg]
